# An automated voxel-based method for calculating the reference value for a brain tumour metabolic index using ^18^F-FDG-PET and ^11^C-methionine PET

**DOI:** 10.1007/s12149-017-1153-8

**Published:** 2017-02-13

**Authors:** Miwako Takahashi, Tsutomu Soma, Akitake Mukasa, Keitaro Koyama, Takuya Arai, Toshimitsu Momose

**Affiliations:** 1 Division of Nuclear medicine, Department of Radiology, Graduate School of Medicine, The University of Tokyo, 3-1 Hongo 7-Chome, Bunkyo-ku, Tokyo, 113-8655 Japan; 2 QMS Group, Quality Assurance Dept., FUJIFILM RI Pharma Co., Ltd., 14-1 Kyobashi 2-Chome Chuo-ku, Tokyo, 104-0031 Japan; 3 Department of Neurosurgery, Graduate School of Medicine, The University of Tokyo, 3-1 Hongo 7-Chome, Bunkyo-ku, Tokyo, 113-8655 Japan

**Keywords:** Brain tumour, Voxel-based analysis, FDG-PET, MET-PET, Tumour-to-normal ratio

## Abstract

**Objective:**

The tumour-to-normal ratio (T/N) is a representative index reflecting brain tumour activity by ^18^F-fluorodeoxyglucose (FDG) and ^11^C-methionine (MET) PET. We proposed a new automated method of calculating the normal reference value (N-value) for use as the denomination of T/N. This method uses voxel-based analysis of FDG- and MET-PET images. We compared the results of this method with those of the standard region-of-interest (ROI) method.

**Methods:**

Data sets were obtained from 32 patients with newly diagnosed glioma and 13 patients with recurrent brain tumour. Our methods were as follows: (1) FDG-PET and MET-PET images were co-registered. (2) The areas where the FDG uptake was higher than a set threshold were selected. (3) For the corresponding areas of MET-PET images, mode and mean voxel values were calculated as tentative MET N-values. (4) Applying the same coordinates to FDG-PET, the voxel values were averaged and used as tentative FDG N-values. (5) The threshold of FDG-PET and whether to use the mode or the mean voxel values were computationally optimized using learning data sets. (6) Applying the optimal threshold and either the mode or mean, N-values of FDG and MET were finally determined.

**Results:**

N-values determined by our automated method showed excellent agreement with those determined by a manual ROI method (ICC(2,1) > 0.78). These values were significantly correlated with mean manual N-values (p < 0.001).

**Conclusions:**

Our new method shows sufficiently good agreement with the standard method and can provide a more objective metabolic index.

**Electronic supplementary material:**

The online version of this article (doi:10.1007/s12149-017-1153-8) contains supplementary material, which is available to authorized users.

## Introduction

The combination of ^18^F-fluorodeoxyglucoe (FDG) and ^11^C-methionine (MET) has been the most effective PET examination for evaluating brain tumours [1–4]. FDG uptake increases with the degree of malignancy in common brain tumour types [1, 4–7]. However, an uptake of FDG is also seen in the normal cortex, which complicates tumour delineation. In addition, FDG uptake in the normal cortex is altered depending on neuronal activities, which can be affected by various factors, such as subclinical epileptic discharge, tumour invasion, and tissue damage from past treatment. These factors should be considered when identifying the reference area in the normal cortex. MET-PET overcomes these difficulties, because MET distribution in the normal cortex is very low and usually not affected by changes in neuronal activity [8]. Therefore, FDG-PET and MET-PET work in a complementary manner to effectively evaluate brain tumours.

When evaluating tumour metabolism, a visual inspection by nuclear medicine experts is usually sufficient for the diagnosis of tumour malignancy; however, the discrimination of uptake level is limited by it being a qualitative process. Therefore, visual inspection is insufficient as a basis for deciding a new drug’s efficacy or for determining a cut-off value for use in treatment management of patients with similar conditions. Therefore, a more objective measurement method is needed.

Metabolic indices, such as standardized uptake value (SUV), tumour-to-normal ratio (T/N), and their modifications, have been used in the previous studies [1, 3, 9–11]. Among these indices, the T/N ratio is the most frequently used and is more favourable for the evaluation of tumour aggressiveness, which also means that the normal cortex is the most appropriate region to use as a reference when evaluating tumour uptake [1, 9]. Compared with T/N ratio, SUV is more prone to inter-subject variability for factors, such as body composition, given that SUV represents the ratio of tumour activity to average body concentration, which is calculated from injected FDG activity and body weight [12].

The T/N ratio is calculated by dividing the tumour SUV by a reference SUV obtained from the normal cortex. Usually, regions-of-interest (ROIs) are placed on the hottest area of the tumour and on an area that appears to be the normal cortex to determine the tumour value and the normal value, respectively. Although the hottest area of the tumour is uniquely determined in most cases, the normal cortex area may not always be reliably identified by visual inspection because of various factors that affect neuronal activity. This is especially true when determining the normal cortex area from FDG-PET images.

In this study, we propose a new automated method in which the voxels corresponding to the normal cortex are identified using characteristics of both FDG-PET and MET-PET, and in which the normal reference values (N-values) are calculated through voxel-based analysis. This method was developed assuming that FDG uptake is relatively high in the normal brain cortex, and that the tumour extent on MET-PET does not exceed more than half of the brain cortex area in most clinical settings. The combination of these characteristics allows the identification of the voxels corresponding to the normal cortex in both of FDG and MET-PET images. If this method is validated, it may provide a more objective index for clinical use.

## Materials and methods

### Patients

We identified 45 patients who underwent both FDG-PET and MET-PET for the evaluation of brain tumour in our department between Mar 2009 and Sep 2014. The pathological diagnosis was performed according to the 2007 World Health Organization guidelines. Thirty-two of these 45 patients (21 men, 11 women; mean age 48 ± 15 years) had untreated primary glioma: 11 with glioblastoma; 12 with anaplastic glioma (8 astrocytoma, 3 oligodendroglioma, and 1 oligoastrocytoma); and 1 with pilocytic astrocytoma. Thirteen of these 45 patients (9 men, 4 women; mean age 54 ± 14 years) experienced recurrence of brain tumour after surgery: 5 with anaplastic glioma (2 astrocytoma, 2 oligodendroglioma, and 1 central neurocytoma); 1 with lung cancer metastasis; and 2 with anaplastic meningioma. We divided the patients into three groups. Group 1 consisted of 20 patients who were randomly selected from the patients with untreated primary glioma, group 2 consisted of the remaining 12 untreated patients, and group 3 consisted of all 13 patients with recurrent brain tumour. The data obtained from group 1 were used as the learning data set of the automated method. The data obtained from groups 2 and 3 were used for the validation of this method. Written informed consent was obtained from all patients. This retrospective study was approved by the institutional review board at our hospital.

### PET/CT protocol

The patients fasted for at least 5 h prior to FDG-PET imaging. The patients rested in the supine position with an eye mask in a quiet PET room to minimize the confounding factors of environmental noises. A 296-MBq (8 mCi) dose of FDG was injected intravenously, and emission scans were obtained 45 min later in three-dimensional mode for 10 min using a PET/CT scanner (Aquiduo, Toshiba Medical System, Otawara, Japan). Photon attenuation correction was performed using a low-dose CT scan. The PET scanner contained 24,336 lutetium oxyorthosilicate crystals in 39 detector rings and had an axial field of view of 16.2 cm, and 82 transverse slices of 2.0-mm thickness. The intrinsic full width at half-maximum (FWHM) spatial resolution at the centre of the field of view was 4.3 mm, and the FWHM axial resolution was 4.7 mm. PET images were reconstructed using Fourier rebinning ordered subset expectation maximization iterative reconstruction, with 2 iterations and 8 subsets, and a 4-mm FWHM Gaussian filter was applied. The data were collected in a 128 × 128 × 41 matrix with a voxel size of 2.0 × 2.0 × 4.0 mm.

For MET-PET imaging, a 740-MBq (20 mCi) dose of MET was injected intravenously, and a 10-min emission scan was started 30 min after the injection. The PET/CT scanner and image reconstruction protocols were the same as the protocols used for FDG-PET imaging.

To conveniently analyse PET images, all voxel values from PET images were normalized to SUV using patient body weight (g), injected radioactivity (Bq/ml), and a cross-calibration factor (Bq/cps), assuming a specific gravity of 1 g/ml.

### Manual ROI-based method

Three experienced nuclear medicine physicians participated as operators in this study. Each of the operators separately placed four circular ROIs with 10-mm diameters on the axial FDG-PET images and MET-PET images manually. These were then compared side-by-side. On the basis of visual inspection, operators placed ROIs in the hemisphere contralateral to the tumour in areas that appeared to be normal grey matter of the superior frontal area and the parietal lobe at the centrum semiovale level, as well as in the inferior frontal area and the temporal lobe at the striatum level. MRI images were also compared with PET images as needed. Each of the three operators calculated a manual N-value by averaging the four ROI measurements. The resulting three N-values were then averaged to produce the “mean manual N-value” used in this study.

### Automated voxel-based method

**Fig. 1:**
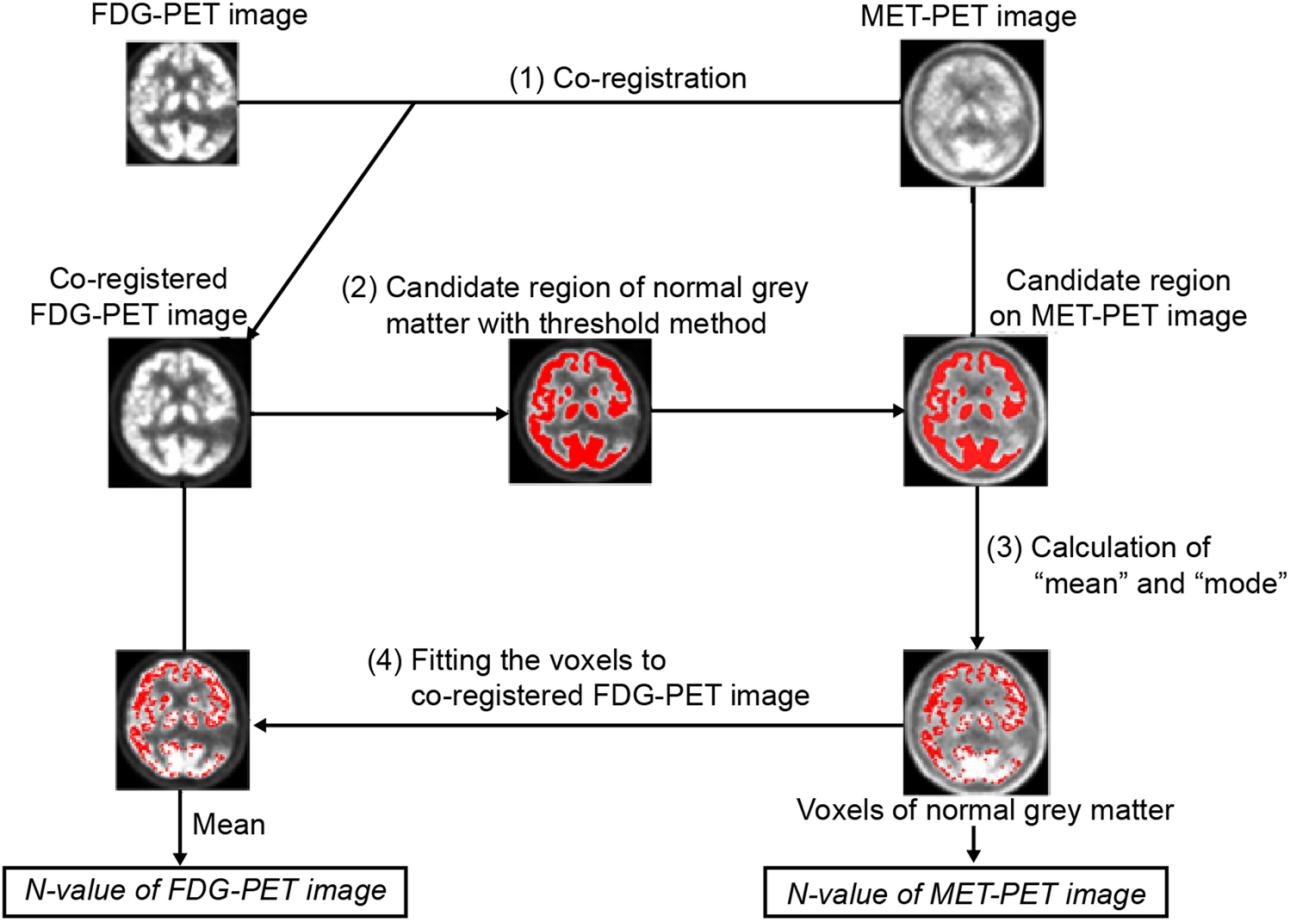
Flowchart of image processes for calculating normal brain cortex value (N-value). For steps 2 and 3, the threshold and whether to use “mean” or “mode” were determined in the optimization step which is not shown in this flowchart. The goal of this computation method is to be able to calculate an appropriate N-value

We developed an automated voxel-based method to determine the N-value for the T/N index. This method was programmed using statistical parametric mapping 8 (SPM8) and MATLAB version R2014a (MathWorks Inc., Natick, MA, USA). The method consisted of four image processing steps and one optimization step. A flowchart of the image processing steps is shown in Fig. 1. In the first step, FDG-PET images were intra-subjectively co-registered to MET-PET images using a normalized mutual information method in SPM8. Co-registration was visually verified by ensuring anatomical agreement between MET-PET and co-registered FDG-PET using the overlay and the crossbar function of MRIcro (http://www.mricro.com). In the second step, a candidate region of normal grey matter was selected from the co-registered FDG-PET as one that had a voxel value higher than a determined optimal threshold. The optimization method for determining this threshold is described below. In the third step, mean and mode MET-PET voxel values from the previously selected normal grey areas were calculated as tentative MET N-values. To calculate mode, histogram bin size was set as 0.1 intervals of SUV. Whether to use the mean or the mode as the parameter in our method was also determined using the optimization method described below. Tentative FDG N-values were calculated by averaging the voxel values that corresponded to the same area as was used to obtain the tentative MET N-values. The most optimal conditions, as determined by the optimization step, were then applied to obtain the final N-values for both MET and FDG.

In the optimization step, we used the data from group 1 to decide two parameters: the threshold value of FDG-PET and whether to use the mean or the mode voxel values from MET-PET. Tentative N-values from the automated voxel-based method were computed by combining either the mean values or the mode values with thresholds ranging from 1.0 to 3.0 times the global mean of FDG-PET in increments of 0.1. The global mean of FDG-PET as calculated after eliminating the voxels outside the brain by masking out values that were less than or equal to one-eighth of the mean total voxel value of the original image. Using these tentative N-values of each subject by three operators with manual method and those with automated method, intraclass correlation coefficients with a two-way random-effects model (ICC(2,1)) were calculated and the optimal parameters were determined by maximizing ICC value.

### Statistical analysis for validation of the automated method using groups 2 and 3

Our automated voxel-based method, which used parameters determined by an optimization process, was applied to the patient imaging data from groups 2 and 3 for validation. To test the reliability of the 3 operator determined manual N-values and the automated N-value, ICC(2,1) values were calculated [13]. An ICC ranging from 0.81 to 0.99 is considered to show a substantial agreement [14]. Pearson’s correlation coefficients were calculated to ascertain the linear association between the automated N-value and the mean manual N-value. In addition, paired *t-*tests were performed to determine the significance of the differences between the results of the automated and manual method, and a Bland–Altman plot was used to identify systemic differences. All statistical tests were two-tailed, and *p* < 0.05 was set as the threshold for statistical significance. All analyses were performed using SPSS 20.0 (IBM, Armonk, NY, USA).

## Results

### Optimization of the parameters

**Fig. 2:**
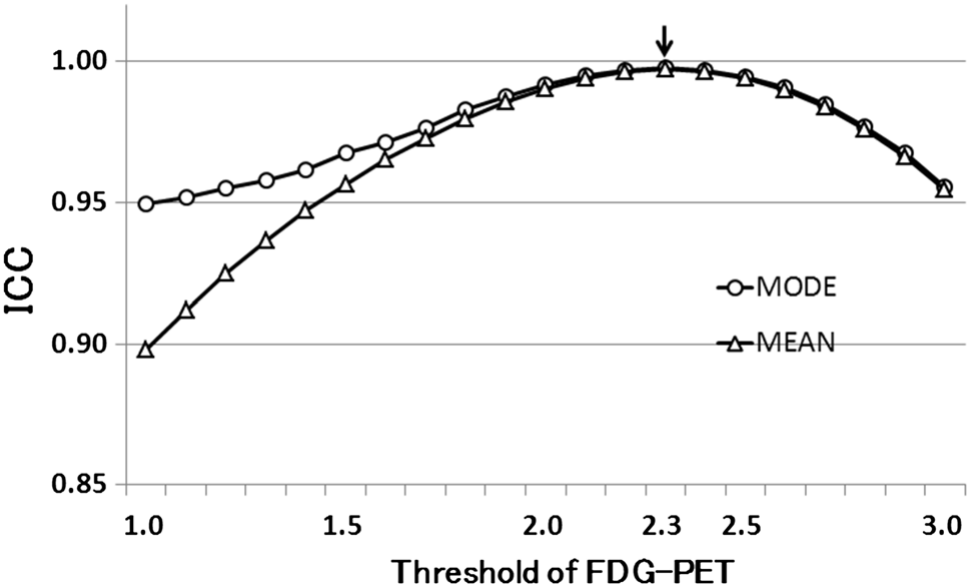
Changes of ICC by threshold value in the optimization step. The *X-axis* shows the threshold values based on the global mean of FDG-PET. Mode refers to the most frequent MET voxel value and mean refers to the average value of MET voxels within the area selected by the threshold method. The ICC is maximum at the threshold 2.3 for the mode curve (*arrow*)

Using the data from patient group 1, ICC value reached the maximum value at a threshold of 2.3 of the FDG-PET and the mod values of MET-PET (Fig. 2).

### Validation of the automated method

Co-registration of FDG- and MET-PET was successfully achieved in all of our patients without the need for manual modification.

**Fig. 3:**
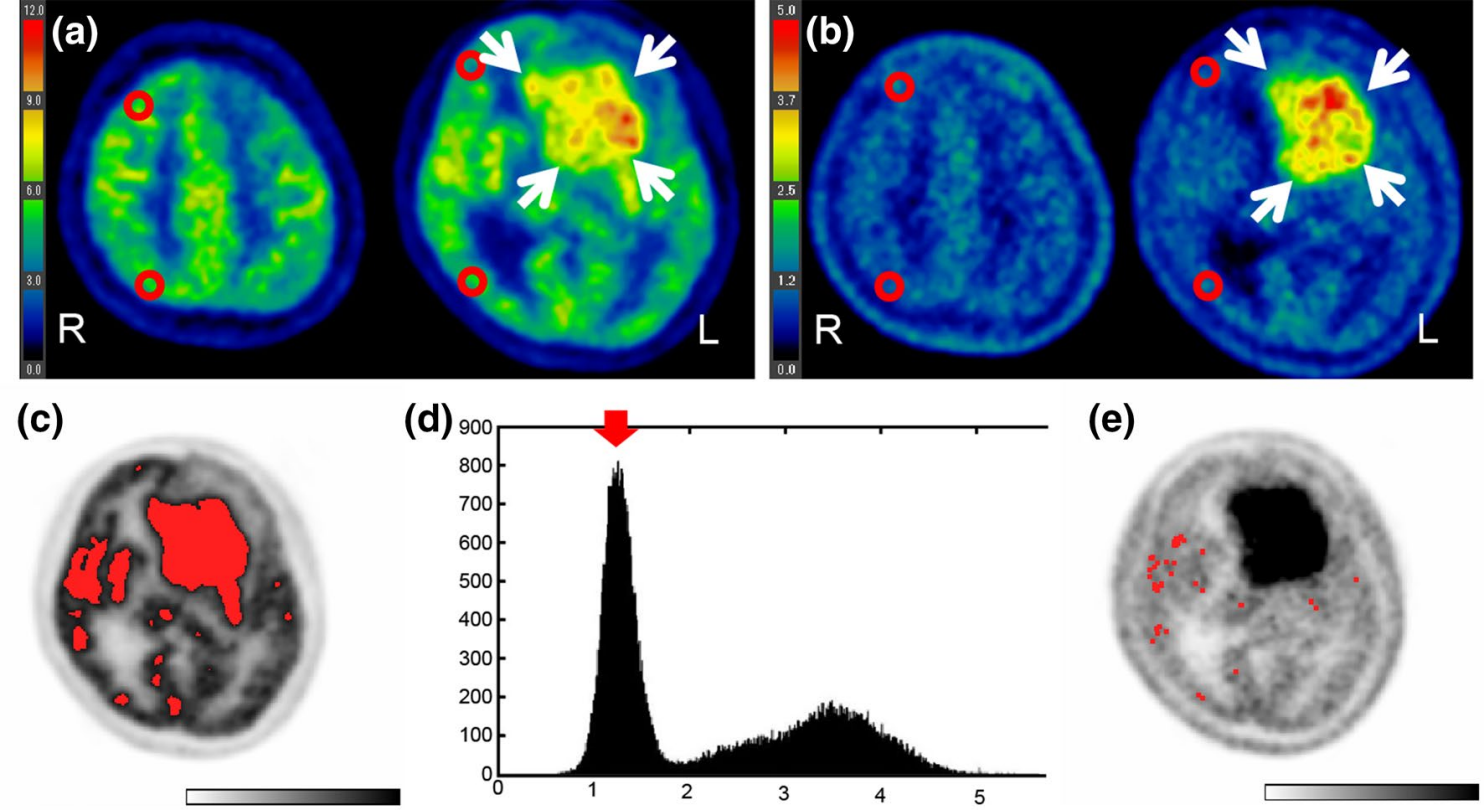
Representative images and data from a patient in group 2. **a, b** Four red circles show the ROIs that were placed manually at the centrum semiovale level and at the striatum level on FDG-PET (**a**) and MET-PET (**b**). A brain tumour with a high uptake of FDG and MET is located in the left frontal lobe (*arrows*). **c** Representative slice of co-registered FDG-PET. The *red area* shows the candidate region for normal grey matter determined using the FDG threshold method, but the FDG-avid tumour is still included. **d** Histogram of all MET voxel values in the area selected with the FDG threshold method. The *Y-axis* represents the number of voxels, and the *X-axis* represents voxel value (SUV). The left peak (*arrow*) is the most frequent voxel value from MET-PET, i.e., the mode used in this study. The right peak mainly corresponds to tumour. **e** Representative slice of MET-PET, on which the finally selected voxels are shown in *red*. Each red voxel is magnified by 9 (3 × 3) to facilitate visualization

**Fig. 4:**
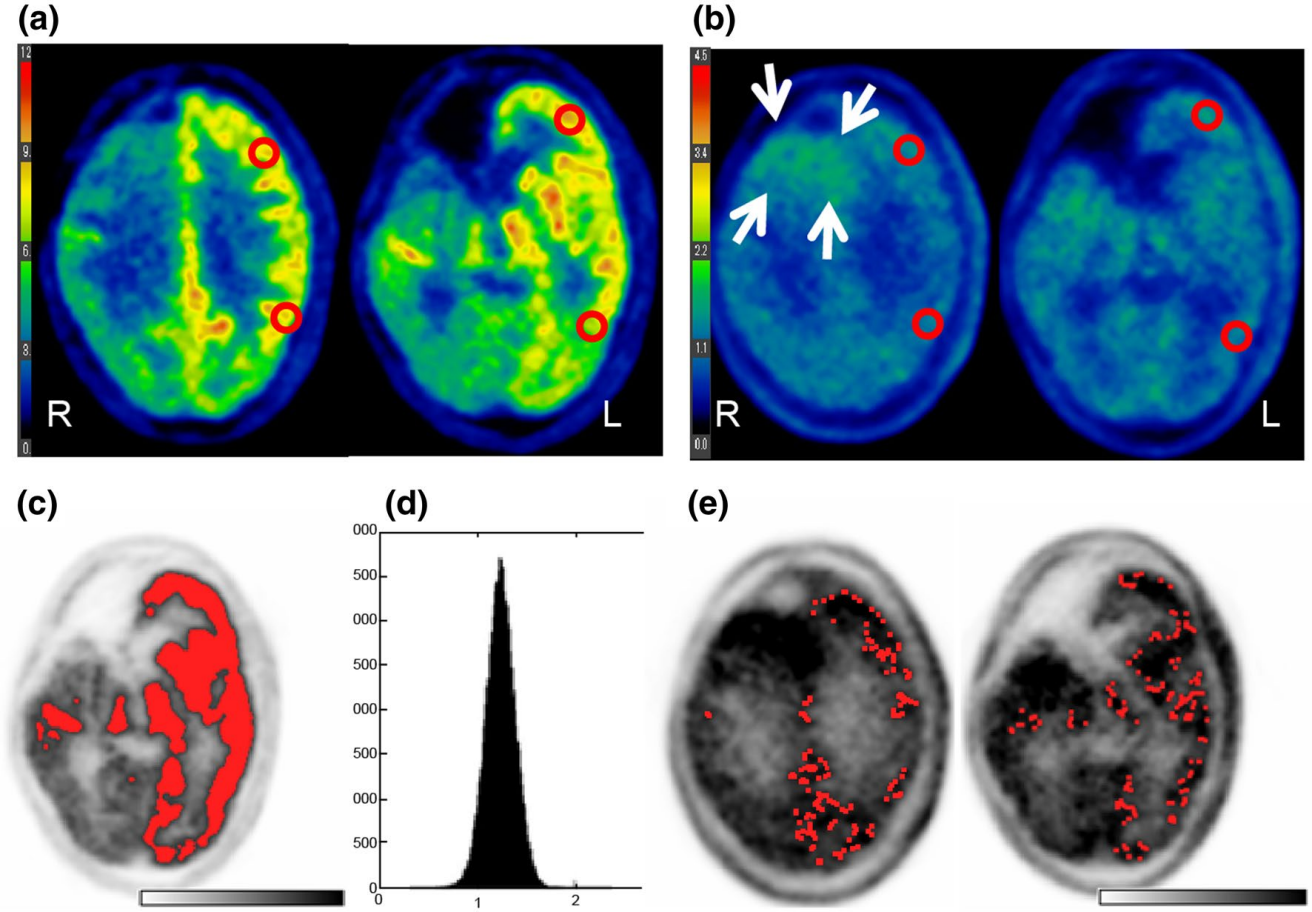
Representative images and data from a patient in group 3. **a, b** Manually placed ROIs at the centrum semiovale level and at the striatum level on FDG-PET (**a**) and MET-PET (**b**). Brain tumour is not distinctive on FDG-PET but can be somewhat visualized in the posterior area of the resection cavity on MET-PET (*arrows*). **c** Representative slice of co-registered FDG-PET. The red area shows the candidate region for normal grey matter determined using the method. **d** Histogram of all MET voxel values from normal grey matter area selected by the FDG threshold method. The *Y-axis* represents the number of voxels, and the *X-axis* represents voxel value (SUV). The peak is the most frequent voxel values from MET-PET, i.e., the mode used in this study. **e** Representative slice of MET-PET, on which the finally selected voxels are shown in *red*. Each red voxel is magnified by 9 (3 × 3) to facilitate visualization

We checked our method by visually confirming that the selected voxels did not include tumour area and included only visually determined normal grey matter, which was achieved automatically without any human interactions in all patients. Representative images of manual ROI placements and those of processes with the automated voxel-based method are shown in Figs. 3 and 4. An FDG- and MET-avid tumour was visualized in the left frontal lobe of a patient in group 2 (Fig. 3a, b). Using the automated method, the tumour area was successfully excluded (Fig. 3c–e). Figure 4 shows images from a patient in group 3. The FDG uptake in the right hemisphere was decreased probably due to a previous surgery and radiation treatment (Fig. 4a). A recurrent tumour with a slightly increase MET uptake is visible in the posterior area of the resection cavity (Fig. 4b). Through the processes of the automated method (Fig. 4c–d), the abnormally decreased FDG uptake area and the recurrent tumour were successfully excluded (Fig. 4e).

### Statistical analysis for validation

**Fig. 5:**
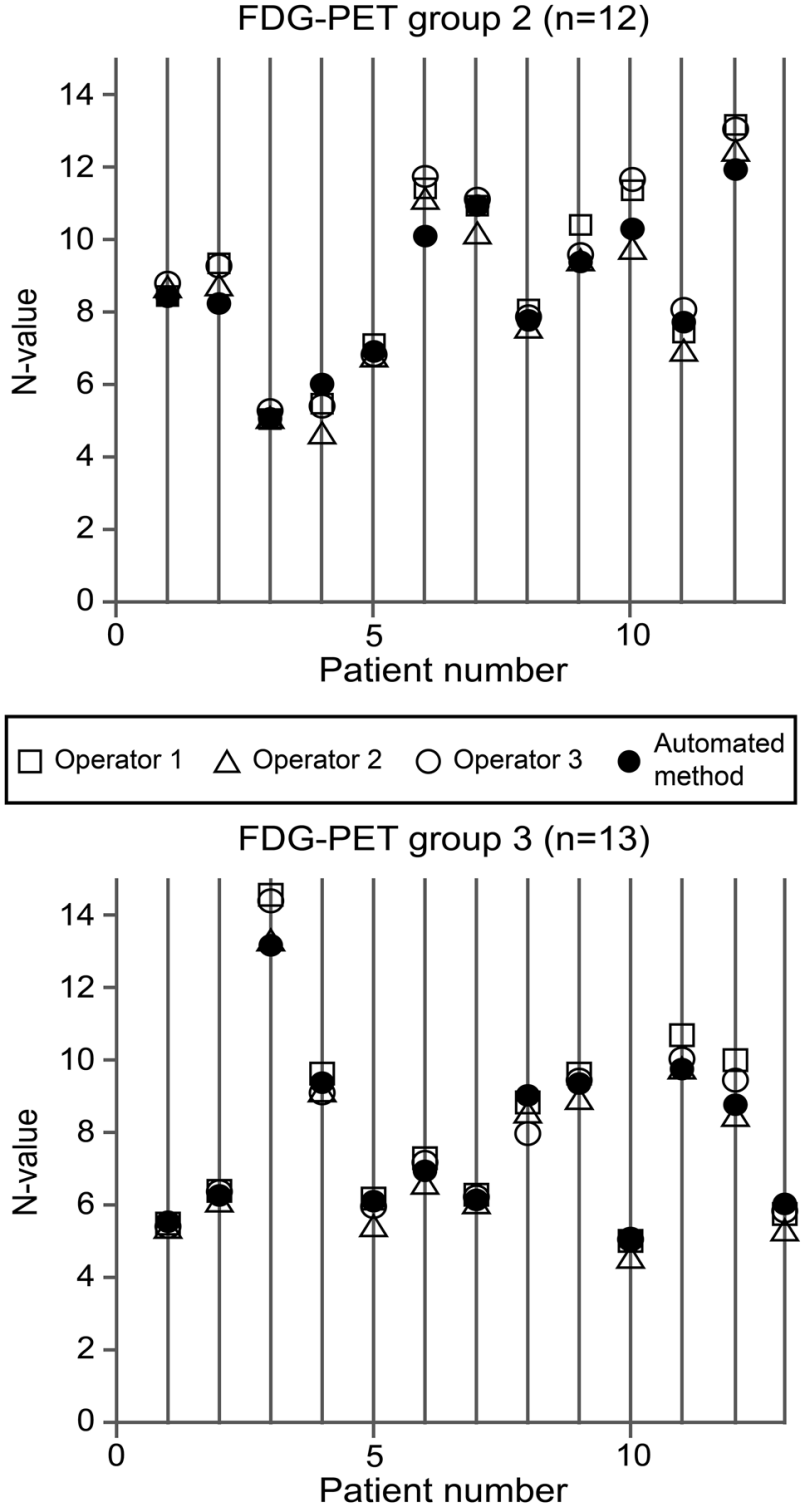
Scatter plots of FDG-PET N-values determined by the automated voxel-based method and those determined by each of three operators using the manual ROI. Group 2 consists of patients with primary glioma and group 3 consists of patients with recurrent brain tumour. The unit of the *y-axes* (N-value) is standardized uptake value

**Fig. 6:**
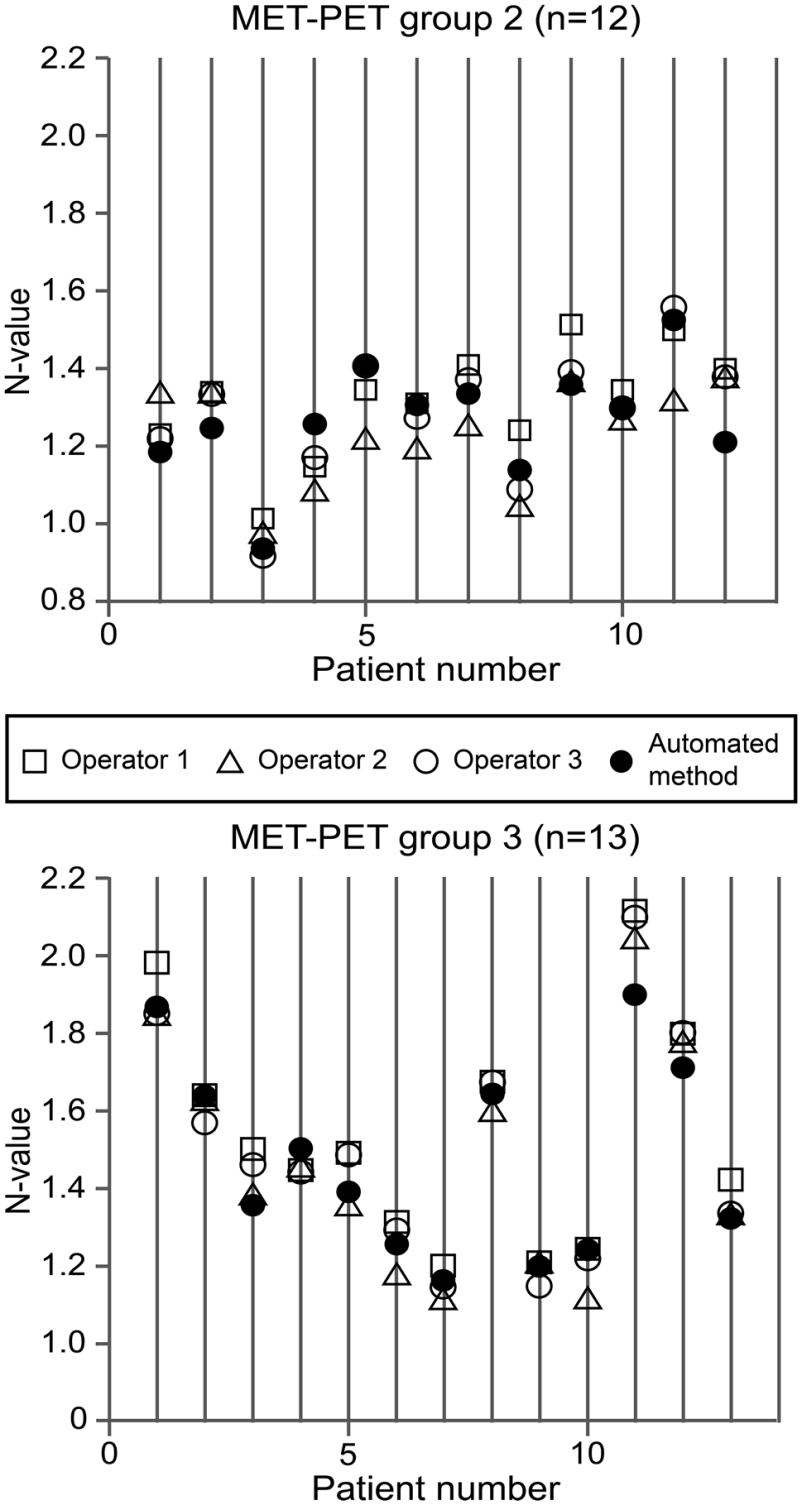
Scatter plots of MET-PET N-values determined by the automated voxel-based method and those determined by each of three operators using the manual ROI method. Group 2 consists of patients with primary glioma and group 3 consists of patients with recurrent brain tumour. The unit of the *y-axes* (N-value) is standardized uptake value

Scatter plots of the N-values obtained from the automated voxel-based method and those obtained from each of the three operators using the manual ROI method are shown in Figs. 5 and 6. The automatically calculated N-values were within the range of the three N-values determined manually in 16/25 (64%) patients for the FDG-PET data, and in 15/25 (60%) patients for MET-PET data. Most of the N-values that were out of the manual range were very close to at least one of the operator N-values. The original N-values and resulting T/N values are presented in the supplementary files (Online Resource 1). Although tumour values were not addressed in this study, they were calculated by placing one circular ROI with a 10-mm diameter on the hottest area of the tumour to demonstrate the differences in T/N ratio.

The results of ICC(2,1) and the corresponding 95% confidential intervals (95%CI) are shown in Table 1. ICC(2,1) of the manual and automated methods was within the range of 0.81 to 0.99 for the FDG values of group 2, and FDG and MET values of group 3, which, therefore, can be considered substantial. In the MET of group 2, ICC(2,1) was slightly low; however, the value of the manual and automated methods was not changed from that of ICC(2,1) across three operators only.

**Table 1:** Intraclass correlation coefficient (ICC) using a two-way random-effects model across N-values determined manually by each of three operators, and across these and N-values determined by the automated voxel-based method

		ICC	95% CI
Group 2			
FDG	3 operators	0.96	0.83–0.99
	3 operators + automated method	0.96	0.87–0.99
MET	3 operators	0.78	0.54–0.93
	3 operators + automated method	0.78	0.56–0.92
Group 3			
FDG	3 operators	0.98	0.88–0.99
	3 operators + automated method	0.98	0.94–0.99
MET	3 operators	0.97	0.82–0.99
	3 operators + automated method	0.96	0.89–0.99

**Fig. 7:**
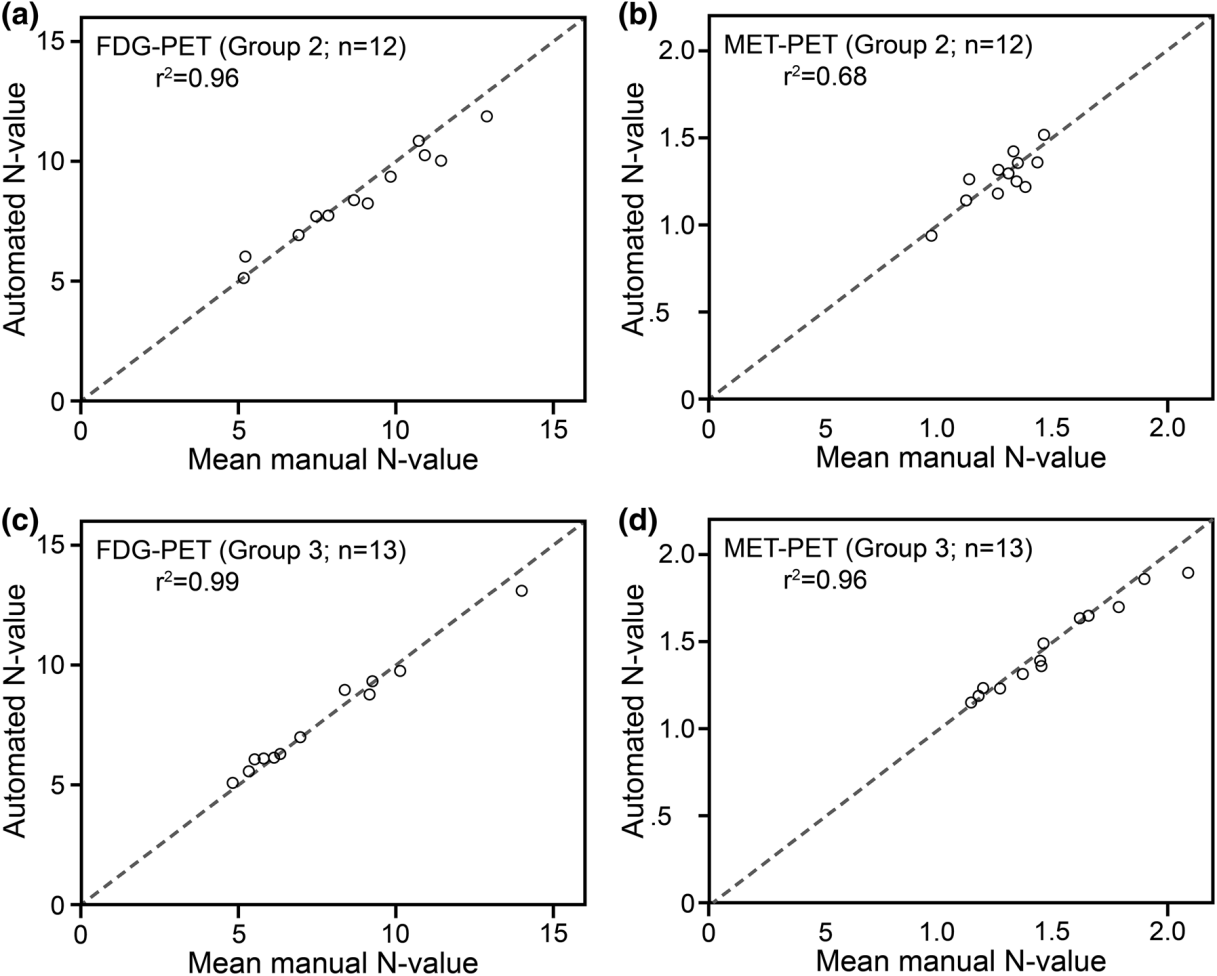
Scatter plots of the N-values determined by the automated voxel-based method and the mean manual N-values. The *x*- and *y-axes* both represent standardized uptake value. The reference dashed line represents the line-of-identity

Scatter plots of the automatically calculated N-values and the mean manual N-values are shown in Fig. 7. Significant linear correlations were found for both validation groups. No significant differences were found using paired t-tests.

**Fig. 8:**
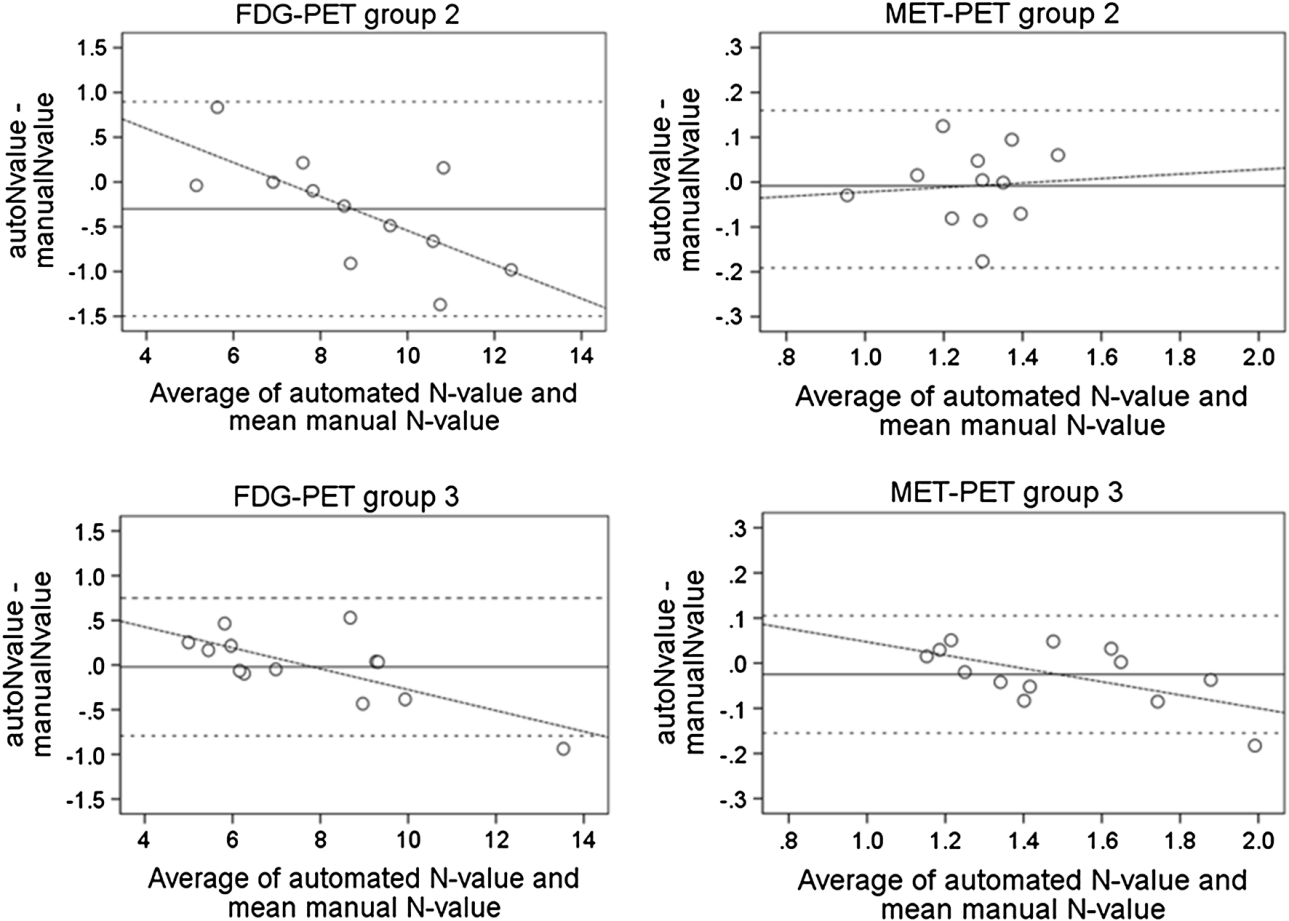
Bland–Altman analysis for identification of systemic difference between the N-values determined by the automated voxel-based method and the mean manual N-value. *Horizontal solid lines* represent the mean difference, *horizontal dot lines* represent mean ± 1.96SD, and *diagonal lines* represent regression lines

Bland–Altman plots are shown in Fig. 8. The mean differences and limits of agreement (mean, mean-1.96 SD, mean + 1.96 SD) are as follows; −0.30, −1.49, +0.90 for FDG-PET from group 2 (Fig. 8a), −0.008, −0.19, +0.16 for MET-PET from group 2 (Fig. 8b), −0.02, −0.79, +0.77 for FDG-PET from group 3 (Fig. 8c), and −0.02, −0.15, +0.11 for MET-PET from group 3 (Fig. 8d). No fixed bias was found. Proportional error was found in the values from MET-PET group 3 and from FDG-PET groups 2 and 3, in which the automated method had a tendency to overestimate the N-value for patients with a low-mean manual N-value and to underestimate the N-value in patients with a high-mean manual N-value. The highest overestimated FDG and MET N-values were 116 and 104% of the mean manual N-values, respectively. The lowest underestimated FDG and MET N-values were 88 and 91% of the mean manual N-values, respectively.

## Discussion

We report the development of a new automated voxel-based method to calculate the N-value required for the T/N index used to evaluate brain tumour. Furthermore, we demonstrate that this method is significantly reliable and that the N-values obtained by this new automated method and the conventional manual ROI-based method are significantly correlated. This new method can be applied regardless of whether a patient has undergone surgical treatment. To our knowledge, this is the first proposal of an automated voxel-based method to calculate normal grey matter values.

FDG-PET and MET-PET are widely used in evaluating brain tumour, but the methods that are used to calculate metabolic indices are not consistent. Most previous studies have employed a ROI-based method for calculating the T/N ratio to evaluate brain tumour, but the procedure for localization of the normal cortex varies among studies. The standard method relies on careful placement of an ROI using visual inspection by an expert. However, this is not a fully objective method and inter-operator variability is unavoidable. Although this variability can be avoided using a fixed ROI template [10, 15], the template method requires morphological normalization and is, therefore, difficult to apply to post-operative patient images. We believe that the new automated method we present in this report may overcome these disadvantages.

Our method relies on two assumptions. First, it relies on the assumption that normal grey matter shows consistently high FDG uptake. The optimal FDG threshold determined by our method can thus extract a sufficient normal cortex region. The second assumption is that the area of brain tumour is not more than half the area of the brain. That is, within the area higher than the optimal FDG threshold, the voxels with the mode or the mean MET value are considered to correspond to the voxels showing normal cortex. This second step can exclude the tumour area if FDG-avid tumour is selected by the FDG-PET threshold method. If these assumptions are not held, such as a situation in which FDG uptake in a large area of grey matter was low due to impaired consciousness and the existence of a large FDG-avid tumour, our method may not have succeeded.

Through an optimization step, we determined that the parameter combination of a threshold of 2.3 of FDG and the mode MET value resulted in optimum N-values. The mode MET value is a more reasonable parameter than the mean MET value, because the latter can be calculated from the voxel values of both normal cortex and tumour area if FDG-avid tumour is selected by the FDG threshold method. Selection of the voxels of the mode MET value successfully excluded the tumour area, as shown in Fig. 3. In this study, median MET value was not included as a parameter, because we believed that it was difficult to determine its clinical meaning or implications and, furthermore, it was less effective in excluding FDG-avid tumours. Nevertheless, we checked the result using the median MET value and found that ICC reached the maximum value at an FDG threshold of 2.3. Although the ICC was the maximum value at a threshold of 2.3, the ICC curve was gradual and close to 1.00 at a threshold ranging from 2.0 to 2.5. Therefore, the threshold may vary around 2.3 depending on the learning data.

When we checked the selected voxels in detail, we observed that the rim of normal grey matter tended to be selected in the cases where FDG accumulation was relatively high, and where FDG accumulation was relatively low, the peak area of the grey matter was selected instead. This phenomenon probably caused the proportional systemic bias seen prominently in the FDG N-values using a Bland–Altman analysis. To determine whether this proportional error is acceptable, we need further studies comparing the automated method with the results of pathological grading and clinical outcome. In this study, the manual method was considered to be the reference standard; however, this does not avoid operator bias. In the fully automated method, it will be helpful in reducing the time needed for the manual calculation and can provide a standard that can be used in multicenter studies.

Our new method was developed without a consideration of any differences in MET distribution throughout the normal brain cortex. MET uptake has been reported to be relatively high in the occipital cortex, cerebellum, and thalamus [15, 16]. In our study, the N-value obtained from the normal reference region determined by our new method was strongly correlated with the results of a standard manual method in which ROIs were placed on the frontal, parietal, and temporal lobes. These regions do not include the areas of high MET uptake reported by the previous studies. Therefore, the development of our automated method was probably not affected by regional differences of normal cortex MET uptake.

A major limitation of our method is the requirement for both FDG and MET-PET. FDG-PET has a role in identifying the candidate region of normal grey matter. Therefore, it may be replaced with MRI when co-registration between MET-PET and MRI is successful using an automated method, and an optimal method of extracting normal grey matter from MRI images is validated. Co-registration can be successful using the mutual information method [17]. A PET-MR device may preclude the need for co-registration processes [18]. Another limitation is the necessity to decide the threshold value of FDG-PET and to decide whether to use the mean or the mode of MET-PET voxel values. These parameters should be optimized using data sets obtained by the same PET protocol.

In conclusion, we have developed a new automated voxel-based method for calculating the N-value of the T/N ratio for the evaluation of brain tumour. Both high reliability and a strong correlation with the conventional manual ROI method were obtained in patients with primary brain tumour and in patients with recurrent tumour after surgery. This is the first automated voxel-based method for providing the N-value needed for calculating a metabolic index. Further investigation will be needed to validate our new method for wider use.

## Electronic supplementary material

Below is the link to the electronic supplementary material.

Supplementary material 1 (XLSX 20 KB)

## References

[1] Kaschten B, Stevenaert A, Sadzot B, Deprez M, Degueldre C, Del Fiore G (1998). Preoperative evaluation of 54 gliomas by PET with fluorine-18-fluorodeoxyglucose and/or carbon-11-methionine. J Nucl Med.

[2] Chung JK, Kim YK, Kim SK, Lee YJ, Paek S, Yeo JS (2002). Usefulness of 11C-methionine PET in the evaluation of brain lesions that are hypo- or isometabolic on 18F-FDG PET. Eur J Nucl Med Mol Imaging.

[3] Van Laere K, Ceyssens S, Van Calenbergh F, de Groot T, Menten J, Flamen P (2005). Direct comparison of 18F-FDG and 11C-methionine PET in suspected recurrence of glioma: sensitivity, inter-observer variability and prognostic value. Eur J Nucl Med Mol Imaging.

[4] Borbely K, Nyary I, Toth M, Ericson K, Gulyas B (2006). Optimization of semi-quantification in metabolic PET studies with 18F-fluorodeoxyglucose and 11C-methionine in the determination of malignancy of gliomas. J Neurol Sci.

[5] Alavi JB, Alavi A, Chawluk J, Kushner M, Powe J, Hickey W (1988). Positron emission tomography in patients with glioma. A predictor of prognosis. Cancer.

[6] Ogawa T, Inugami A, Hatazawa J, Kanno I, Murakami M, Yasui N (1996). Clinical positron emission tomography for brain tumors: comparison of fludeoxyglucose F 18 and L-methyl-11C-methionine. AJNR Am J Neuroradiol.

[7] Borgwardt L, Hojgaard L, Carstensen H, Laursen H, Nowak M, Thomsen C (2005). Increased fluorine-18 2-fluoro-2-deoxy-D-glucose (FDG) uptake in childhood CNS tumors is correlated with malignancy grade: a study with FDG positron emission tomography/magnetic resonance imaging coregistration and image fusion. J Clin Oncol.

[8] Kajimoto K, Oku N, Kimura Y, Kato H, Tanaka MR, Kanai Y (2007). Crossed cerebellar diaschisis: a positron emission tomography study with L-[methyl-11C]methionine and 2-deoxy-2-[18F]fluoro-D-glucose. Ann Nucl Med.

[9] Utriainen M, Metsahonkala L, Salmi TT, Utriainen T, Kalimo H, Pihko H (2002). Metabolic characterization of childhood brain tumors: comparison of 18F-fluorodeoxyglucose and 11C-methionine positron emission tomography. Cancer.

[10] Prieto E, Marti-Climent JM, Dominguez-Prado I, Garrastachu P, Diez-Valle R, Tejada S (2011). Voxel-based analysis of dual-time-point 18F-FDG PET images for brain tumor identification and delineation. J Nucl Med.

[11] Okochi Y, Nihashi T, Fujii M, Kato K, Okada Y, Ando Y (2014). Clinical use of (11)C-methionine and (18)F-FDG-PET for germinoma in central nervous system. Ann Nucl Med.

[12] Keyes JW, Jr. SUV (1995). standard uptake or silly useless value?. J Nucl Med.

[13] Shrout PE, Fleiss JL (1979). Intraclass correlations: uses in assessing rater reliability. Psychol Bull.

[14] Shrout PE (1998). Measurement reliability and agreement in psychiatry. Stat Methods Med Res.

[15] Coope DJ, Cizek J, Eggers C, Vollmar S, Heiss WD, Herholz K (2007). Evaluation of primary brain tumors using 11C-methionine PET with reference to a normal methionine uptake map. J Nucl Med.

[16] Uda T, Tsuyuguchi N, Terakawa Y, Takami T, Ohata K (2010). Evaluation of the accumulation of (11)C-methionine with standardized uptake value in the normal brain. J Nucl Med.

[17] Galldiks N, Ullrich R, Schroeter M, Fink GR, Jacobs AH, Kracht LW (2010). Volumetry of [(11)C]-methionine PET uptake and MRI contrast enhancement in patients with recurrent glioblastoma multiforme. Eur J Nucl Med Mol Imaging.

[18] Boss A, Bisdas S, Kolb A, Hofmann M, Ernemann U, Claussen CD (2010). Hybrid PET/MRI of intracranial masses: initial experiences and comparison to PET/CT. J Nucl Med.

